# Hearing in action; auditory properties of neurons in the red nucleus of alert primates

**DOI:** 10.3389/fnins.2014.00105

**Published:** 2014-05-13

**Authors:** Jonathan M. Lovell, Judith Mylius, Henning Scheich, Michael Brosch

**Affiliations:** ^1^Special Lab for Primate Neurobiology, Leibniz Institute for NeurobiologyMagdeburg, Germany; ^2^Deutsches Zentrum für Neurodegenerative ErkrankungenMagdeburg, Germany

**Keywords:** red nucleus, auditory, primate, electrophysiology, neuron

## Abstract

The response of neurons in the Red Nucleus pars magnocellularis (RNm) to both tone bursts and electrical stimulation were observed in three cynomolgus monkeys (*Macaca fascicularis*), in a series of studies primarily designed to characterize the influence of the dopaminergic ventral midbrain on auditory processing. Compared to its role in motor behavior, little is known about the sensory response properties of neurons in the red nucleus (RN); particularly those concerning the auditory modality. Sites in the RN were recognized by observing electrically evoked body movements characteristic for this deep brain structure. In this study we applied brief monopolar electrical stimulation to 118 deep brain sites at a maximum intensity of 200 μA, thus evoking minimal body movements. Auditory sensitivity of RN neurons was analyzed more thoroughly at 15 sites, with the majority exhibiting broad tuning curves and phase locking up to 1.03 kHz. Since the RN appears to receive inputs from a very early stage of the ascending auditory system, our results suggest that sounds can modify the motor control exerted by this brain nucleus. At selected locations, we also tested for the presence of functional connections between the RN and the auditory cortex by inserting additional microelectrodes into the auditory cortex and investigating how action potentials and local field potentials (LFPs) were affected by electrical stimulation of the RN.

## Introduction

The Red Nucleus (RN) or *nucleus ruber* lies in the rostral midbrain and derives its name from the high concentration of iron-containing pigments within its cellular structure (Hernandez, 1931). The RN is comprised of two subnuclei, the rostral pars parvocellularis (Oka and Jinnai, 1978; Onodera and Hicks, 2009), and the smaller caudal pars magnocellularis (Yamaguchi and Goto, 2006). Gibson et al. (1985) found that in rhesus monkeys, the magnocellular RN controls the onset, velocity, and duration of specific upper limb movements, with electrical stimulation causing discrete contractions of limb muscles in the shoulder, elbow, wrist, and digits.

Compared to its role in motor behavior little is known about sensory response properties of neurons in the RN, particularly those concerning the auditory modality. In chloralose-anesthetized cats, neurons in the rostral RN have been found to exhibit short-latency responses to clicks and tones (Massion and Albe-Fessard, 1963; Irvine, 1980). Tuning curves are generally very broad, and neurons receive inputs from both ears and are sensitive to interaural time and level differences (Shinkarenko, 1984; Shinkarenko et al., 1985). Bratus et al. (1981) recorded evoked potentials from the magnocellular RN in anesthetized cats, and showed that the response latency varies from 3.5 to 10.5 ms, depending on the intensity of the auditory stimulation. It has been hypothesized that the RN is a component of the subcortical path for reflexes connected with turning the ear in the direction of sound (Courville, 1968), and that it is a part of the complex pathways concerned with the animal's postural and defensive responses to acoustic stimulation (Martin and Dom, 1970).

Here we report auditory response properties of the RN in alert macaque monkeys. These data were obtained in a series of studies that were primarily designed to characterize effects of the dopaminergic ventral midbrain on auditory processing. The auditory responses in the RN attracted our attention because of their potential role in audiomotor interactions, i.e., its possible involvement in guiding movement-related reactions to sounds, and its involvement in monitoring the sounds that are evoked by movement.

In these experiments, we moved microelectrodes along tracks oriented approximately in a mediolateral direction, through different deep brain structures toward RN. Sites in the RN were recognized by observing electrically evoked body movements characteristic for this deep brain structure. At the same time, the electrodes served to record action potentials and local field potentials (LFPs) from the RN, and to characterize neuronal responses to clicks and pure tones. At selected locations, we also tested for the presence of functional connections between the RN and the auditory cortex by inserting additional microelectrodes into the auditory cortex and analysing how action potentials and LFPs were affected by electrical stimulation of the RN.

## Materials and methods

The subjects used in this experiment were three adult cynomolgus monkeys (*Macaca fascicularis*); monkey E (male, 5.5 kg) monkey W (male, 6.2 kg), and monkey M (female, 4 kg). Each monkey was fitted with a head holder and a recording chamber (18 mm diameter), which was positioned in the right (monkeys E and M) and the left (monkey W) temporal regions of the skull. Details of the surgery are given elsewhere (Brosch and Scheich, 2008). Experiments were conducted in a sound-attenuated double-walled room. During the experiments the monkeys were seated in a primate chair and their heads were fixed to allow for acute recordings. All experiments were carried out under approval of the animal care and ethics committee of the State Sachsen-Anhalt (No. 28-14 42502/2-806 IfN) and in accordance with the guidelines for animal experimentation of the European Communities Council Directive (86/609/EEC).

Micro-fiber electrodes were used for both electrical stimulation and recording neuronal activity. The electrodes were constructed from a single fiber with a 25 μm tungsten and iridium core, insulated with quartz glass to a total diameter of 80 μm, with tips sharpened to a point using an ultrafine diamond grinder. The impedance of each electrode was tested and found to be in the ranges of 0.5 MΩ for recording electrodes to less than 25 kΩ for low impedance stimulation electrodes. The electrodes were then fashioned to fit in the micro-drive and were able to extend over 40 mm through the macaque brain. Electrodes were advanced remotely into the brain from outside of the sound-attenuating room using a seven-channel microdrive (System Eckhorn, Thomas Recording).

Through these electrodes, we simultaneously recorded multiunit activity using a band-pass filter set between 1 to 7 kHz, and LFPs with a band-pass of 0.1 to 250 Hz, using the systems SUA-02 and LFP-03 (Thomas Recording). All signals were fed into an AD data-acquisition system (32-channel Alpha-Map, Alpha-Omega). The LFP sampling rate was 658 Hz, and for multiunit activity it was 50 kHz. We only stored time stamps of multiunit activity when spike amplitudes exceeded noise thresholds.

**Figure 1:**
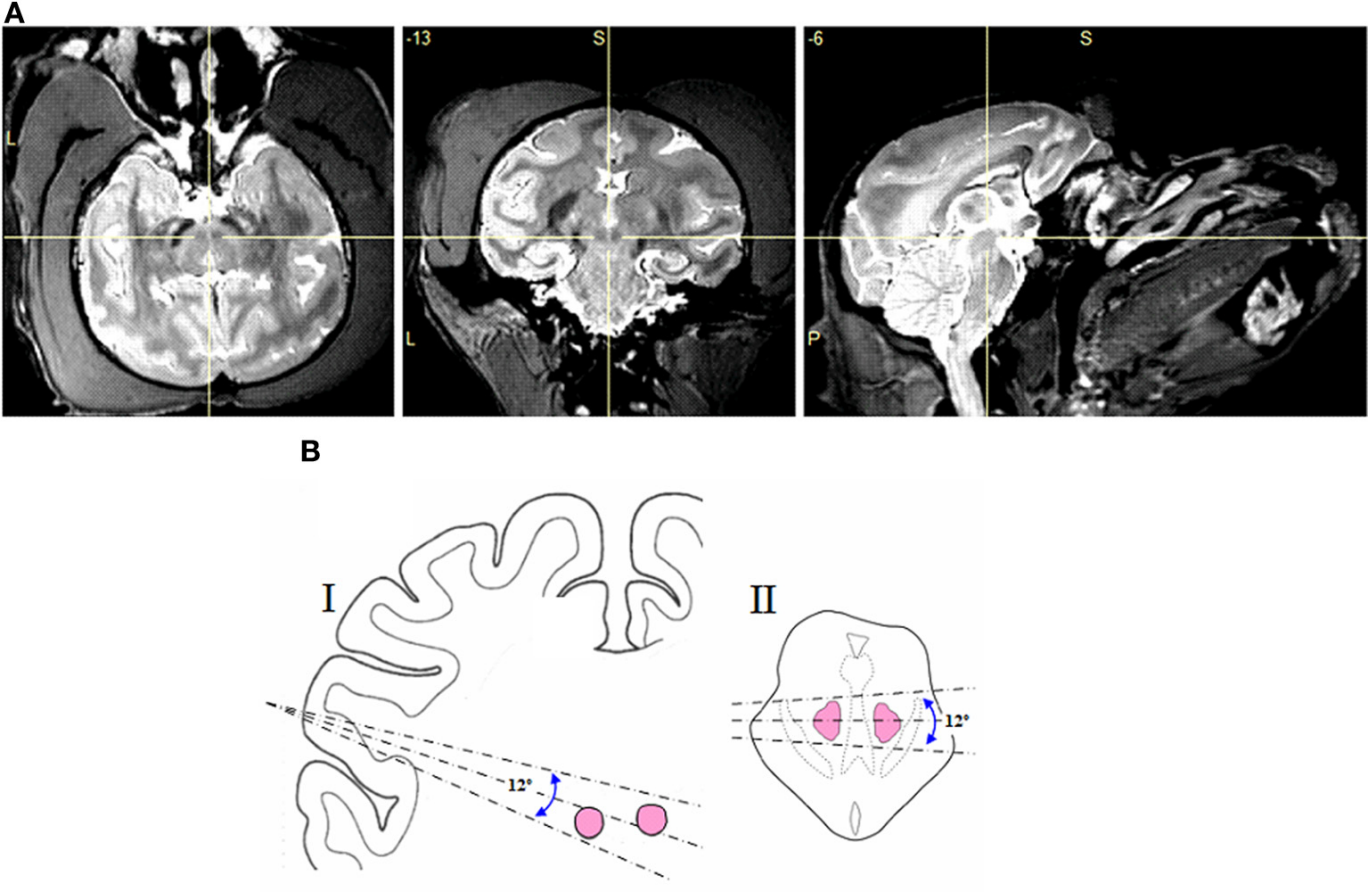
**(A)** MRI images of the macaque brain (monkey E) in axial, coronal, and sagittal views, with the cross hair showing the location of the red nucleus. **(B)** Range of electrode angles used in the sagittal **(I)** and axial **(II)** planes.

Initially, MRI images were used to plan electrode trajectories toward the RN. As can be seen in Figure 1A, the iron rich pigmentation within the RN makes this structure highly visible when imaged using MRI. The temporal location of the recording chambers restricted the electrode trajectories to those shown in Figure 1B Thus, electrodes were inserted into the brain at an angle of 18 (±6) degrees from the horizontal plane. Once the electrodes had been advanced to around 19 mm from the surface of the dura membrane, we commenced presentation of electrical and auditory stimulation to the monkey. Finer localization of the two red nuclei was pinpointed by observing upper body movements (e.g., arms, shoulders, face, mouth, and eyelids) in response to the electrical stimulation (note that the monkeys were head fixed and seated in a primate chair, thus limiting the full expression of certain electrically evoked movements). No histological verification of stimulation sites was undertaken because more than one hundred stimulation sites were tested in each monkey and because all monkeys in the current study are still involved in other research projects, following the principles of Replacement, Reduction, and Refinement in animal experimentation.

Multiunit activity and LFPs were recorded from the auditory cortex using a 16-channel microdrive (System Eckhorn, Thomas Recording). The microdrive was oriented slightly off the dorsoventral plane (10°). Electrodes entered the cortex between stereotactic coordinates A5 to A9 and D7.5 to D12.5 and subsequently were moved through the parietal cortex into the core fields of the auditory cortex.

Monopolar electrical stimulation was delivered using a Multichannel Systems STG 400-4 stimulator, programmed to generate 50 ms trains of square edged biphasic pulses. These were presented at a frequency of 100 Hz, and a pulse width of 700 μs and at a maximum intensity of 200 μA, to evoke minimal body movements only. We presented acoustic stimulation in the form of clicks and tone bursts. Clicks were produced by a waveform generator (WG1, Tucker-Davis Technologies) and presented every 2 s during each of the electrode tracks. Tone bursts were generated using a computer coupled to an array processor (Tucker-Davis Technologies, Gainesville, FL), which converted the signal from digital to analog at a sample rate of 100 kHz. Each frequency (40 in total) was repeated 10 times, in pseudorandom order, from 0.11 to 27.2 kHz, covering a range of 8 octaves spaced by 0.2 of an octave. The tone burst envelope was 100 ms in duration, ramped by 5 ms with an inter tone interval of 900 ms. The auditory stimulus gain was increased using an amplifier (model A202; Pioneer, Long Beach, CA), then fed to two bilaterally placed free-field loudspeakers (Canton Karat 720.2), positioned 1.1 m from the subject, at intensities not exceeding 65 dB (re. 20 μPa). The sound pressure level was measured with a free-field 1/2′ microphone (40AC, G.R.A.S.), located near to the monkey's head.

Custom written MATLAB (version 2007b, MathWorks, Natick, MA, USA) programs were used for the off-line analyses of multiunit and LFP recordings. In the RN, tuning curves were generated from each multiunit recording of responses to the tone bursts which were presented at 40 different frequencies, as described elsewhere (Brosch et al., 1999). From these tuning curves, we obtained the best frequency, the bandwidth, the first-spike latency, and the last-spike latency. In the auditory cortex, we computed a post-stimulus time histogram (PSTH) with a bin size of 10 ms for each of the multiunit recordings, relative to the onset of electrical stimulation in the RN (≥50 trials). Because the electrical stimulation generated an artifact that lasted maximally 20 ms, this period was excluded from the analysis. A multiunit recording was considered to have responded to the electrical stimulation if the number of discharges within at least two of 30 consecutive 10-ms post-stimulus bins was significantly above the number of discharges immediately before stimulus presentation (Wilcoxon signed rank tests, two-sided). For each of the LFP recordings, evoked potentials were calculated by averaging the LFP relative to the onset of auditory or electrical stimulation. In order to analyse auditory-evoked potentials, the lowest value of the first main trough of the waveform was used to indicate the response latency and frequency bandwidth.

## Results

**Figure 2:**
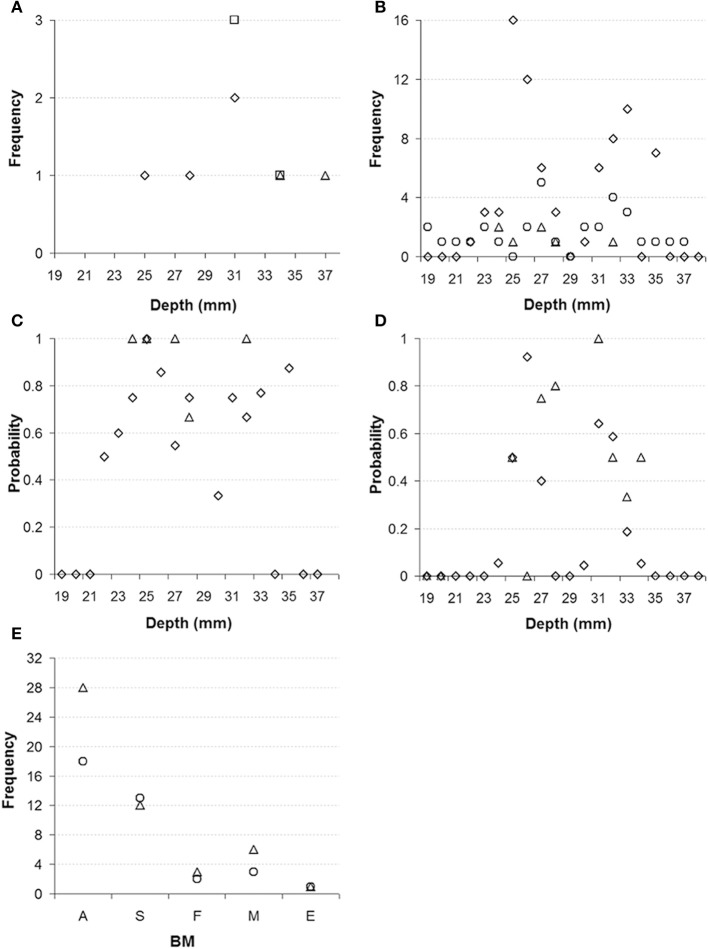
**(A)** Representative electrode track through the left and right red nucleus in monkey E. Diamonds represent where electrical stimulation evoked upper body movements (indicative of red nucleus), squares represent responses to auditory stimulation, and triangles represent locations where no responses to electrical stimulation were observed. **(B)** Frequency of sites where electrical stimulation evoked upper body movement for all electrode tracks (diamonds monkey E, triangles monkey W) and frequency of unresponsive sites (circles). **(C)** Probability of sites with electrical evoked upper body movements (diamonds monkey E, triangles monkey W). **(D)** Probability of sites with auditory responses (diamonds monkey E, triangles monkey W). **(E)** Category and frequency of the main body movements evoked by the electrical stimulation of the left and right RN (monkeys E and W combined). A, arm; S, shoulder; F, face; M, mouth; E, eye (left nucleus: circles, right nucleus: triangles).

In this study we applied brief electrical stimulation to 118 deep brain sites in two monkeys (monkey E and monkey W). Figure 2A shows the results of a representative track through the red nuclei, following the center dashed line from Figure 1B. At 86 of these sites (76 in monkey E and 10 in monkey W) we could evoke upper body movements that are characteristic for the RN. Figures 2B,C show the frequency and probability of evoked upper body movements and no responses to the electrical stimulation for all tracks in monkeys E and W. At the remaining sites, no such movements were observed. Upper body and face movements were exclusively observed in two clusters between 22 and 28 mm and between 30 and 35 mm from the dura surface, which is in good correspondence to the location of the two red nuclei in both hemispheres and relative to published brain atlases (see Figure 1B).

When we recorded action potentials from these 118 stimulation sites, we found that 18 sites (14 in monkey E and 4 in monkey W) responded to the click stimulation, which was presented every 2 s to the monkeys. With one exception, the auditory responses were found exclusively at the 86 sites where electrical stimulation evoked upper body movements. Thus, auditory responses were found at 19.7% of the sites in the RN. We also tested the click stimulation at another 335 sites (312 in monkey E and 23 in monkey W) at which electrical stimulation was not applied directly to reduce the number of electrical stimulations delivered to the monkeys, but which fell within 200 μm from a site from which upper body and face movements were elicited. Of these sites, most are presumed to be located in the RN; 67 sites (19.5%; 55 in monkey E and 12 in monkey W) did respond to the click stimulation. Figure 2D shows the probability for finding click responses for all of the 335 sites in and around the RN. Figure 2E shows the category and frequency of the main body movements evoked by electrical stimulation of the left and right RN.

Indication for auditory responses in the RN was also obtained at 23 of 51 sites in a third monkey (monkey M). From this monkey, neuronal recordings were obtained from a brain region that corresponded to that tested in monkeys E and W, and following the same stereotactic coordinates used on the two monkeys. Electrical stimulation was omitted so long as the electrode was estimated to be within the RN, in order to reserve electrical stimulation exclusively for locations within the ventral tegmental area where the monkey would receive brain stimulation reward for bar pressing. In this experiment, the presence of auditory responses around the estimated depth of the RN was actually used as a landmark to determine the position of the proximal ventral tegmental area.

**Figure 3:**
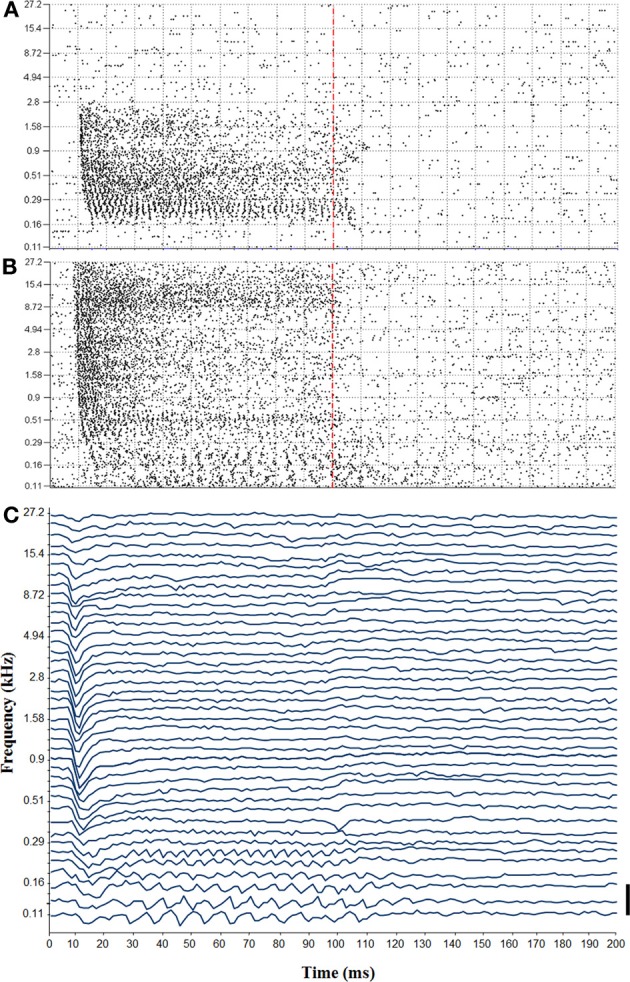
**(A)** Tuning curve recorded from the left red nucleus. Vertical dashed line indicates the end of the tone bursts. **(B)** Tuning curve from the right red nucleus. Note the temporal coding showing phase locking to low frequency tones. **(C)** Auditory-evoked potential recorded in responses to tone bursts from the right red nucleus (bar = 100 μV).

Auditory sensitivity of RN neurons was analyzed more thoroughly at 15 sites (12 in monkey E and 3 in monkey W), where strong auditory responses to clicks were observed and from where upper body movements could be electrically evoked, or which fell within 200 μm from such a site. To achieve this, we presented 400 tone bursts over a range of frequencies from 0.11 to 27.2 kHz. Figures 3A,B shows spike responses to the tonal stimuli from two representative sites.

The auditory sensitivity of the RN was also detected in the LFPs that were recorded in parallel with the spikes. Figure 3C shows auditory-evoked potentials that were obtained from the same site at which the tuning curve was obtained from the spike responses shown in Figure 3B. We noticed that, similar to the spike responses, auditory-evoked potentials were phase locked to low stimulus frequencies during the full duration of the tones (note that our filter setting did not allow for us to reliably detect frequency following responses above 250 Hz). For higher stimulus frequencies, by contrast, auditory-evoked potentials consisted only of an initial, negative going wave that occurred about 10 ms after stimulus onset, but which was not followed by any later wave. Auditory potentials were evoked from a frequency range that was similarly as wide as the range that elicited multiunit responses.

**Figure 4:**
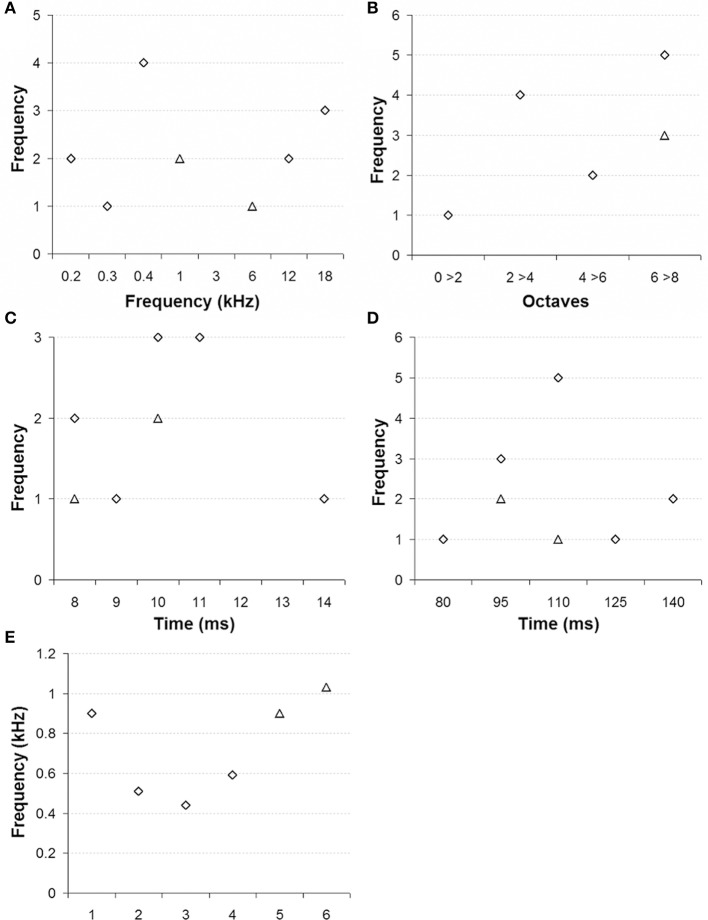
**Tonal selectivity of multiunit activity in red nucleus**. **(A)** Best frequency, **(B)** Tuning curve bandwidths in octaves, **(C)** Latency of the first spike, **(D)** Latency of the last spike, and **(E)** Maximum frequency of phase locking (monkey E: diamonds, monkey W: triangles).

In our sample, RN neurons had best frequencies that were distributed between 0.16 and 17.75 kHz (median 0.9 kHz; Figure 4A) and generally exhibited quite broad tuning curves (median 7.2 octaves; Figure 4B). Responses started between 9 to 17 ms post-tone onset (median 11 ms; Figure 4C) and lasted, in most cases, for the full duration of the stimulus sound (Figure 4D). We also noted that at 6 sites (4 in monkey E and 2 in monkey W) the responses were partially phase locked to the waveform of the tested sounds. The upper limit of phase locking ranged between 0.44 and 1.03 kHz (median 0.9 kHz; Figure 4E).

**Figure 5:**
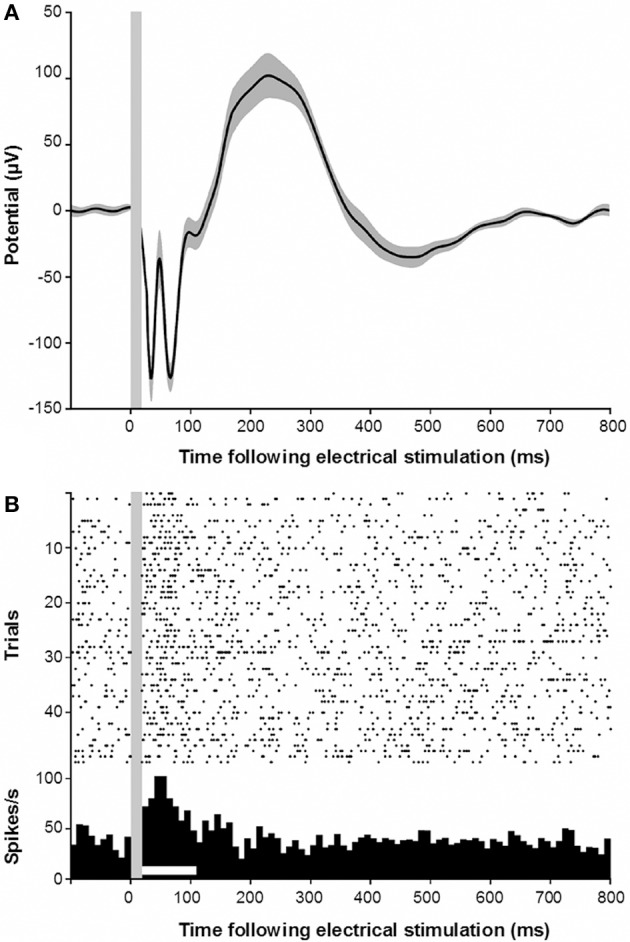
**(A)** Electrically evoked potential recorded in the auditory cortex following electrical stimulation of the red nucleus. Gray shading indicates s.e.m. Gray vertical bar indicates the stimulus artifact. **(B)** Electrically evoked multiunit responses in the auditory cortex following electrical stimulation of the red nucleus.

Our experimental approach also allowed us to demonstrate a functional connection from the RN to the primary auditory cortex. To this end, we applied monopolar electrical stimulation to the RN and performed microelectrode recordings of LFPs and spikes from the auditory cortex. Analysis of 204 stimulation/recording pairs (monkeys E and W) revealed that such stimulation always resulted in an electrically evoked potential in the auditory cortex, which is similar to the representative evoked potentials shown in Figure 5A. The earliest negative trough of the evoked waveform occurred around 34 ms post-electrical stimulation. This was rapidly followed by a positive peak and a second negative trough, followed by a slow positive deflection ending after about 400 ms post-stimulation. At a few sites (*n* = 15, 7.4%) in the auditory cortex, electrical stimulation in the RN even resulted in an elevated firing of the neurons at a latency of around 50 ms and a duration of 90 ms (Figure 5B).

## Discussion

This is the first time that auditory responses have been recorded from the RN of primates, and reveals that groups of neurons that control upper body movement also respond to acoustic signals. The auditory sensitivity observed here is generally in good agreement with that previously observed in chloralose-anesthetized cats (Massion and Albe-Fessard, 1963; Irvine, 1980; Bratus et al., 1981; Shinkarenko, 1984; Shinkarenko et al., 1985). Thus, neurons in the RN respond with short latencies, to a broad range of tone frequencies, can phase lock their responses to quite high tone frequencies, and respond to the inputs from the two ears, resulting in some selectivity for localizing sound sources. These auditory properties suggest that the RN receives inputs from the non-lemniscal part of the auditory brainstem, even though such projections appear not be present (Massion, 1967; Mylius et al., 2013). A striking difference to the previous studies is the percentage of sites in the RN at which auditory responses were found. Whereas in all cat studies more than 90% of neurons were found to respond to clicks or tones, in the monkey RN such responses were only found in around 20% of the sites. These differences may reflect methodological differences in defining the borders of the RN (e.g., anatomy vs. electrically evoked movements, or anesthetized vs. alert). They may even reflect species differences, such as the importance of pinnae movements for sound source localization. Thus, it is possible that the RN may be under less auditory influence in primates than in lower mammals, and perhaps reflects a diminished importance of the RN in motor functions, particularly in primates.

The existence of responses to auditory and electrical stimulation in these brainstem nuclei is of considerable use, especially as a landmark when targeting proximal “harder to detect” structures, such as the ventral tegmental area. Our study also suggests a functional connection from the RN to the auditory cortex. Electrical stimulation of the RN-evoked post-synaptic potentials in the auditory cortex, which were suprathreshold in some neurons, thereby causing them to fire action potentials. The latter finding together with the short response latency of ~30 ms, suggests the presence of a projection from the RN to the auditory cortex that has a slow conduction velocity in the order of 1 mm/ms although the existence of this pathway still needs to be anatomically demonstrated.

Our findings corroborate previous accounts that the RN provides a place for audiomotor interactions (Courville, 1968; Martin and Dom, 1970; Shinkarenko, 1984; Shinkarenko et al., 1985). Since the RN appears to receive inputs from a very early stage of the ascending auditory system, our results suggest that sounds can modify the motor control exerted by this brain nucleus. Our finding of a functional connection from the RN to the auditory cortex suggests that neuronal activity controlling motor behavior might also affect auditory processing in the auditory system. This pathway might even provide a source for motor-related activity in the auditory cortex (Brosch et al., 2005).

### Conflict of interest statement

The authors declare that the research was conducted in the absence of any commercial or financial relationships that could be construed as a potential conflict of interest.
